# Methamphetamine facilitates pulmonary and splenic tissue injury and reduces T cell infiltration in C57BL/6 mice after antigenic challenge

**DOI:** 10.1038/s41598-021-87728-4

**Published:** 2021-04-15

**Authors:** Adriana C. Hernandez-Santini, Anum N. Mitha, Daniela Chow, Mohamed F. Hamed, Azad L. Gucwa, Valerie Vaval, Luis R. Martinez

**Affiliations:** 1 Department of Biology, University of Puerto Rico-Ponce, Ponce, PR USA; 2 Department of Biomedical Sciences, NYIT College of Osteopathic Medicine, New York Institute of Technology, Old Westbury, NY USA; 3 Department of Biological Sciences, The Border Biomedical Research Center, The University of Texas at El Paso, El Paso, TX USA; 4 Department of Oral Biology, University of Florida College of Dentistry, 1395 Center Drive, Room DG-48, P.O. Box 100424, Gainesville, FL 32610 USA; 5 Department of Pathology, Faculty of Veterinary Medicine, Mansoura University, Mansoura, Egypt; 6 Department of Biology, Farmingdale State College, Farmingdale, NY USA; 7 Department of Biomedical Sciences, Long Island University, C. W. Post, Brookville, NY USA

**Keywords:** Biological techniques, Cell biology, Immunology, Physiology

## Abstract

Methamphetamine (METH) is a strong addictive central nervous system stimulant. METH abuse can alter biological processes and immune functions necessary for host defense. The acquisition and transmission of HIV, hepatitis, and other communicable diseases are possible serious infectious consequences of METH use. METH also accumulates extensively in major organs. Despite METH being a major public health and safety problem globally, there are limited studies addressing the impact of this popular recreational psychostimulant on tissue adaptive immune responses after exposure to T cell dependent [ovalbumin (OVA)] and independent [lipopolysaccharide (LPS)] antigens. We hypothesized that METH administration causes pulmonary and splenic tissue alterations and reduces T cell responses to OVA and LPS in vivo, suggesting the increased susceptibility of users to infection. Using a murine model of METH administration, we showed that METH causes tissue injury, apoptosis, and alters helper and cytotoxic T cell recruitment in antigen challenged mice. METH also reduces the expression and distribution of CD3 and CD28 molecules on the surface of human Jurkat T cells. In addition, METH decreases the production of IL-2 in these T-like cells, suggesting a negative impact on T lymphocyte activation and proliferation. Our findings demonstrate the pleotropic effects of METH on cell-mediated immunity. These alterations have notable implications on tissue homeostasis and the capacity of the host to respond to infection.

## Introduction

Methamphetamine (METH) is a highly addictive illicit drug and a potent psychostimulant that has become a significant worldwide public health problem due to its recreational popularity^1^. In the United States (U.S.), METH is consumed by 1.6 million people every year. Its consumption has substantially increased 7 to 8 times in the last decade^2^. METH increases the release of dopamine in reward regions of the brain reinforcing the user’s craving for consumption and repetitive behavior, leading to addiction^3–5^. METH abuse contributes to violent crime and 15% of all drug overdose deaths in the U.S., with half of those deaths due to an opioid^6^. METH abuse costs the U.S. healthcare system approximately $30 billion per year having detrimental psychological, medical, and social effects in its users^7^. METH use can cause memory loss, aggression, psychotic behavior, cardiovascular damage, and severe dental problems. In addition, individuals are at an increased risk of acquiring HIV^8^, hepatitis^9^, tuberculosis^10^, and other transmissible diseases^11^ as consequences of METH use. These infectious diseases can spread via contaminated needles, syringes, and other equipment used by multiple people who inject METH^8^.

The high accumulation of METH in most organs in the body adversely impacts adaptive immunological responses^12^, which may contribute to a higher rate and more rapid progression of certain infections in drug users^8,10^. METH compromises cell-mediated immunity and considerably reduces T cells in rodents via apoptosis^13,14^. METH administration also causes mitochondrial oxidative damage and effector defects in human T cells^15^. In fact, METH exposure alters CD4^+^ helper and CD8^+^ cytotoxic T lymphocyte populations resulting in increased HIV proliferation in infected individuals^16–18^. CD4^+^ T cells recognize peptides presented on MHC class II molecules on antigen (Ag) presenting cells (APCs) and play a central role in modulating adaptive immune responses, whereas, CD8^+^ T lymphocytes recognize peptides presented by MHC class I molecules, which are found in all nucleated cells. Cytotoxic T cells are important for combating viral infections. The T cell receptor (TCR) on both, CD4^+^ and CD8^+^ T cells, binds to the Ag loaded MHC on the surface of the APC^19^. This triggers the initial activation of the T cells. However, T cells require several secondary signals to become activated and respond to infection. For instance, in the case of CD4^+^ T cells, the first of these secondary signals is provided by CD28. This molecule on the T cell binds to one of two molecules on the APC [B7.1 (CD80) or B7.2 (CD86)] and initiates T cell proliferation.

Given that METH abundantly accumulates in pulmonary and splenic tissues of users and their susceptibility to viral infections, we investigated the impact of this drug on organ architecture and T cell recruitment in the lungs and spleen of C57BL/6 mice after challenge with T-dependent [TD; ovalbumin (OVA)] and T-independent [TI; lipopolysaccharide (LPS)] Ag. Moreover, we explored the effects of METH on the expression and distribution of T cell surface molecules involved in the activation of cell-mediated immunity. We hypothesized that METH administration may result in tissue injury and reduce T cell responses to OVA and LPS in vivo, suggesting the increased susceptibility of users to infection by commonly transmissible viral pathogens. We aimed to demonstrate the pleotropic effects of METH on the adaptive immune response. These alterations may have profound implications on tissue homeostasis and the capacity of METH users to respond to diverse insults, including invading viral pathogens.

## Results

### METH modifies inflammation and induces apoptosis in pulmonary tissue

**Figure 1:**
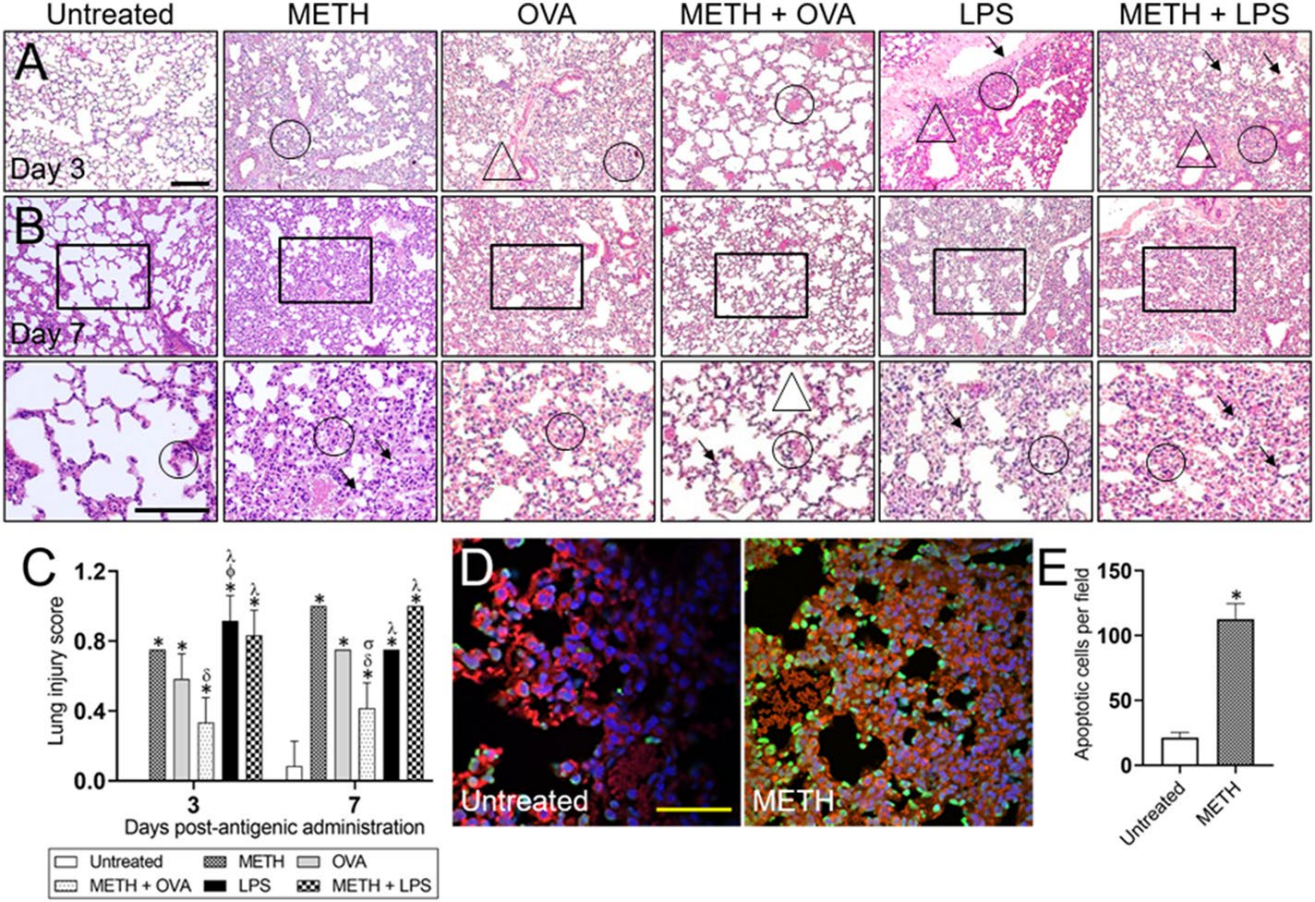
Increasing and prolonged doses of methamphetamine (METH) cause morphological alterations to the pulmonary tissue of C57BL/6 mice. **(A)** Representative 10 × magnification of hematoxylin and eosin (H&E) stained sections of pulmonary tissue excised from untreated, METH, ovalbumin (OVA), METH + OVA, lipopolysaccharide (LPS), and METH + LPS-treated animals after 3 (upper panel) and 7 (middle panel) days post-Ag administration are shown (scale bar, 100 μm). **(B)** Magnified (40 ×) black boxes of lung tissue after 7 days (bottom panel) post-Ag administration (scale bar, 100 μm). For panels **A** and **B**, circles, triangles, and arrows indicate specific morphological changes explained in the result section. **(C)** The lung injury score (LIS) was evaluated blindly by two independent investigators. Each bar indicates the average score for 5 fields per image per investigator and error bars indicate standard deviations (STDEV). Bars without error bars denote conditions with no variability in LIS. Symbols (*, ϕ, δ, σ, and λ) indicate *P* value significance (*P* < 0.05) calculated using analysis of variance (ANOVA) and adjusted by use of the Tukey’s post-hoc analysis. *, ϕ, and λ indicate significantly higher LIS than in the untreated, OVA, and METH + OVA-treated groups, respectively. δ and σ indicate significantly lower LIS than in the METH and OVA-treated groups, respectively. The lungs of untreated mice did not show any injury after 3 days post-Ag challenge, therefore, a bar for the group (LIS = 0) is not present. **(D)** Lung tissue excised from untreated and METH-treated mice (*n* = 5 per group) after administration (21 days) were analyzed for apoptosis by fluorescent microscopy after being stained with TUNEL (green nuclei); representative images are presented. The nuclei of viable cells were stained with dapi (blue). **(E)** Counts of apoptotic cells per field of lung tissue from untreated and METH-treated mice were determined using fluorescent microscopy. Bars indicate the average number of apoptotic cells (*n* = 10 images; 10 fields per image; 2 images per mouse; 5 mice per condition) and error bars indicate STDEV. An asterisk (*) indicates *P* value significance (*P* < 0.05) calculated using student’s *t*-test analysis.

The respiratory system has the highest uptake of METH in users^12^ making them prone to acquire pulmonary infection such as tuberculosis^10^ or HIV-secondary related infections^20^. Thus, we investigated the effect of METH on the lung inflammatory responses and architecture of C57BL/6 mice challenged with OVA and LPS 3- and 7-days post-Ag challenge (Fig. 1). On day 3 post-Ag sensitization, the lungs of untreated animals exhibited normal pulmonary architecture (Fig. 1A). METH-treated mice displayed marked proliferation of interstitial tissue with pulmonary atelectasis or partial collapse (Fig. 1A; circle) of certain areas of the lung. OVA-treated mice demonstrated widespread coagulative necrosis of pulmonary tissue (Fig. 1A; circle) and marginal localization of leukocytes (Fig. 1A; triangle) on the pulmonary blood vessels. Respiratory tissue of rodents treated with METH and challenged with OVA evinced patent pulmonary alveoli and congestion of interstitial capillaries without white blood infiltrates (Fig. 1A; circle). LPS-treated mice demonstrated a widespread congestion of the interstitial capillaries with neutrophilic recruitment into the alveolar lumen (Fig. 1; circle), alveolar collapse (Fig. 1; triangle), and slit-like holes in the capillary basal laminae (Fig. 1A; arrow). In addition, METH-treated and LPS-sensitized animals displayed considerable neutrophilic recruitment into the pulmonary alveoli (Fig. 1A; circle) and hyperplasia of type II pneumocytes (Fig. 1A; arrows) with some alveolar bronchiolization (Fig. 1A; triangle).

On day 7 post-Ag challenge, untreated animals showed neutrophilic plugging of the interstitial capillaries (Fig. 1B; bottom panel at higher magnification; circle) and slight expansion of alveolar walls with no exudate in the alveolar lumen. METH-treated mice exhibited extensive coagulative necrosis (Fig. 1B; circle) and an intense infiltration of macrophagic, epithelioid, and polymorphic cells (Fig. 1B; arrows). OVA-treated rodents maintained their pulmonary architecture but showed a widespread coagulative necrosis characterized by a minimal inflammatory exudate (Fig. 1B; circle). Notably, METH + OVA animals displayed normal pulmonary alveoli with minor emphysematous alterations (Fig. 1; triangle), slight congestion of their interstitial capillaries (Fig. 1B; circle), and limited neutrophilic infiltrates (Fig. 1B; arrow). Mice treated with LPS evinced coagulative necrosis of pulmonary tissue (Fig. 1; circle) in addition to slight infiltration of phagocytic cells (Fig. 1B; arrow). METH + LPS-treated rodents also showed pulmonary tissue with extensive coagulative necrosis (Fig. 1B; circle) and cellular infiltration of macrophage and epithelioid origin (Fig. 1B; arrows).

We used a lung injury score (LIS) to determine the murine pulmonary tissue damage after METH injection for 3 weeks using increasing doses (2.5 to 10 mg/Kg) alone or followed by OVA or LPS challenge (Fig. 1C). We observed significant LIS in mice from all the conditions compared to the untreated animals 3- and 7-days post-Ag administration (*P* < 0.05). On day 3 post-Ag sensitization, METH + OVA-treated mice showed lower LIS than animals in the METH-treated group (*P* < 0.05). LPS-challenged mice demonstrated higher LIS relative to rodents in the OVA and METH + OVA-treated groups (*P* < 0.05). Additionally, METH + LPS-treated animals showed higher pulmonary damage than METH + OVA-treated mice (*P* < 0.05). On day 7 post-Ag challenge, METH + OVA mice displayed lower LIS than animals in the METH- and OVA-treated groups (*P* < 0.05). Similarly, both LPS-sensitized groups exhibited higher pulmonary tissue damage than the METH + OVA rodents (*P* < 0.05). Interestingly, we did not experience variations of LIS in all the experimental groups after 3- and 7-days post-Ag administration.

Since we observed that METH administration causes substantial damage to the respiratory system, we investigated the impact of this substance of abuse on pulmonary cellular apoptosis using TUNEL and fluorescent microscopy (Fig. 1D-E). METH-treated mice demonstrated higher number of apoptotic cells (green) in pulmonary tissue than untreated mice (Fig. 1D). Counts of apoptotic cells per field confirmed the presence of significant apoptotic cells in the lungs of METH-treated rodents relative those of control animals (Fig. 1E). Our findings indicate that prolonged METH administration alters the mammalian inflammatory response of the respiratory system and cause considerable pneumocyte death and damage of pulmonary tissue. Importantly, recruitment of T cells via OVA stimulation results in a reduction of neutrophils in METH-damaged lung tissue.

### METH causes splenic tissue alterations and enhances splenocyte apoptosis in mice

**Figure 2:**
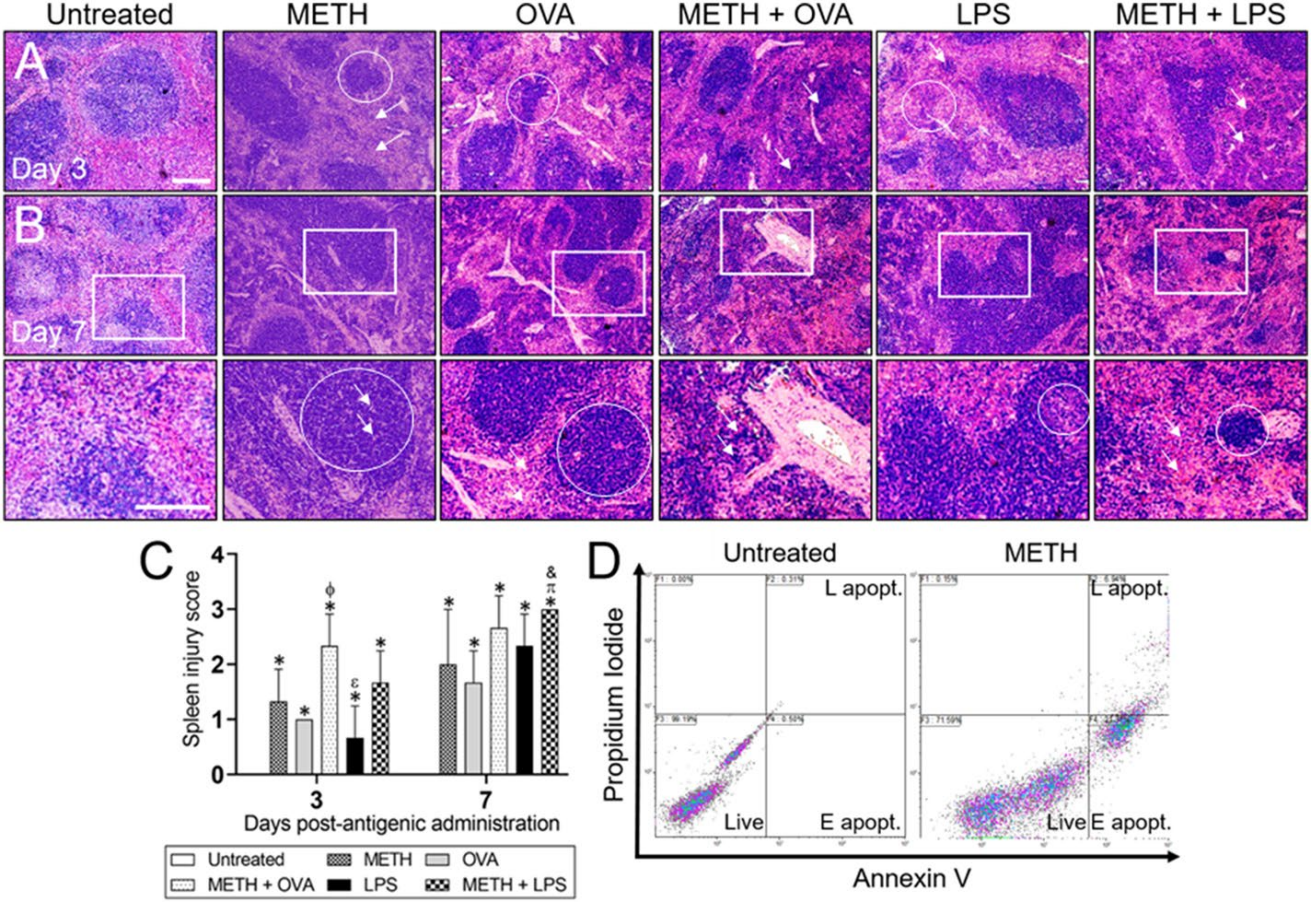
Increasing and prolonged doses of METH damage the spleen of C57BL/6 mice. **(A)** Representative 10 × magnification of H&E stained sections of splenic tissue excised from untreated, METH, OVA, METH + OVA, LPS, and METH + LPS-treated mice after 3 (upper panel) and 7 (middle panel) days post-Ag administration are shown (scale bar, 100 μm). **(B)** Magnified (40 ×) white boxes of splenic tissue after 7 days (lower panel) post-Ag administration (scale bar, 100 μm). For panels **A** and **B**, circles and arrows indicate specific morphological changes explained in the result section. **(C)** The spleen injury score (SIS) was evaluated blindly by two independent investigators. Each bar indicates the average score for 5 fields per image per investigator and error bars indicate STDEV. Bars without error bars denote conditions with no variability in SIS. Symbols (*, π, ϕ, &, and ε) indicate *P* value significance (*P* < 0.05) calculated using ANOVA and adjusted by use of the Tukey’s post-hoc analysis. *, π, ϕ, and & indicate significantly higher SIS than in the untreated, METH, OVA, and LPS-treated groups, respectively. ε indicates significantly lower SIS than in the METH + OVA-treated group. The spleens of untreated mice did not show any injury after 3- and 7-days post-Ag challenge, therefore, a bar for the group (SIS = 0) is not present. **(D)** Splenocytes excised from untreated and METH-treated mice (*n* = 5 per group) were analyzed for apoptosis by flow cytometry after being stained with annexin V-FITC together with propidium iodide (PI); representative plots are presented. The percentages of viable (live), early apoptotic (E apopt.) and late apoptotic (L apopt.) cells are reported.

METH causes immunosuppression and the spleen harbors abundant number of cells and molecules important for the establishment of an effective immune response^21–23^. Therefore, we determine the effects of METH on the spleen anatomical structure and the cellular responses to stimulation with TD and TI Ag (Fig. 2). Histological analyses of splenic tissue 3 days post-Ag administration demonstrated that spleens of untreated mice had normal lymphoid follicles, germinal center, and red pulp (Fig. 2A). In contrast, METH-treated mice exhibited atrophy of lymphoid follicles (Fig. 2A; circle) and increased interfollicular spaces (Fig. 2A; arrows). Rodents challenged with OVA displayed splenic lymphoid necrosis and atrophy of the lymphoid follicles (Fig. 2A; circle). The spleens of METH + OVA mice also showed noticeable lymphoid necrosis with loss of follicular pattern in clusters resembling follicular remnants (Fig. 2A; arrows). LPS-treated animals had spleens with lymphoid necrosis (Fig. 2A; arrow) and expansion of the red pulp at the expense of the white pulp (Fig. 2A; circle). Moreover, mice treated with METH and sensitized with LPS exhibited necrotic splenic tissue with small aggregates of lymphoid remnants instead of a follicular pattern (Fig. 2A; arrows).

On day 7 post-Ag sensitization, the anatomy of the spleen of untreated mice looked normal with germinal centers with dense basophilic granulocytes (Fig. 2B). METH-treated animals denoted visible hyperplasia (Fig. 2B; bottom panel at higher magnification; circle) of the lymphoid follicles with necrosis resembling cribriform-like pattern or pierced tissue with small holes (Fig. 2B; arrows). The spleens of OVA-treated mice showed atrophy of the lymphoid follicles (Fig. 2B; circle) and increased interfollicular spaces (Fig. 2B; arrows). METH-treated rodents challenged with OVA had splenic tissue with severe necrosis of the lymphoid follicle and periarteriolar lymphoid sheath with expansion of red pulp at the expense of white pulp (Fig. 2B; arrows). LPS-challenged mice demonstrated comedo-like necrosis (Fig. 2B; circle) of the splenic lymphoid tissue in the hyperplastic follicle. Furthermore, METH + LPS-treated rodents showed splenic tissue with massive lymphoid necrosis (Fig. 2B; circle) and only remnants of lymphoid follicles. Expansion of red pulp (Fig. 2B; arrows) was also observed in splenic tissue treated with METH + LPS, with a marked decrease in white pulp.

We documented the damage prolonged METH injections with weekly increased doses causes to splenic tissue by implementing a spleen injury score (SIS; Fig. 2C). On days 3 and 7 post-Ag administration, the SIS of mice treated with METH, Ag, or combination was significantly higher than those observed in untreated animals (*P* < 0.05). METH + OVA-treated mice had considerably higher SIS than animals challenged with either OVA or LPS 3 days post-Ag sensitization (*P* < 0.05). Also, rodents treated with METH and challenged with LPS evinced substantial SIS increased relative to mice treated with METH or LPS alone 7 days post-Ag administration (*P* < 0.05). In contrast to lung tissue, spleens excised from mice treated with METH, Ag, or combination displayed a time-dependent damage increased (Fig. 2C).

We examined whether increasing doses and prolonged METH administration facilitates splenocyte apoptosis by flow cytometry (Fig. 2D) using annexin V-fluorescein isothiocyanate (FITC; green) and propidium iodine (PI; red). Viable cells with intact membranes exclude PI, whereas the membranes of dead and damaged cells are permeable to PI. Flow cytometry of splenic tissue homogenates validated that untreated cells demonstrated 99.19% viability. Spleens of mice treated with METH displayed 71.59% viable, 21.32% early and 6.94% late apoptotic cells. Thus, splenocytes from METH-treated mice exhibited significantly higher apoptosis than those isolated from untreated rodents (*P* < 0.05). Together, our data revealed that METH causes substantial anatomical changes to the spleen and contribute to splenocyte cell death in vivo.

### METH modifies CD4^+^ cell recruitment into the lung and splenic tissue of C57BL/6 mice after OVA or LPS challenge

**Figure 3:**
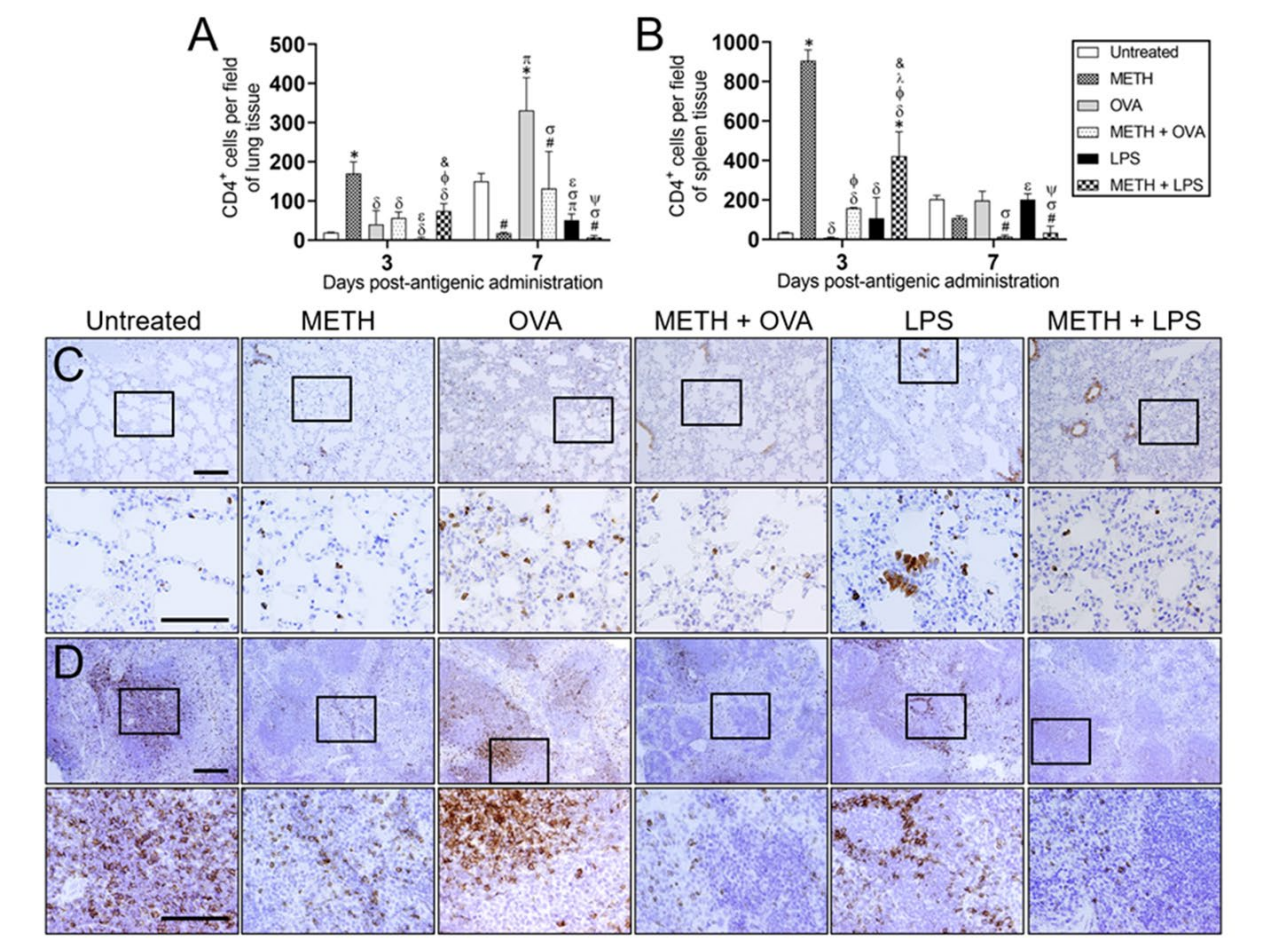
METH inhibits CD4^+^ cell recruitment into the lung and splenic tissue of C57BL/6 mice after OVA or LPS challenge. Counts of helper T (CD4^+^) cells per 0.1 g of **(A)** lung and **(B)** splenic tissue of C57BL/6 mice (*n* = 5 mice per condition per day) 3- and 7-days post-METH and OVA/LPS administration were analyzed by microscopic counts. Bars indicate the average number of CD4^+^ cells (*n* = 5 mice per condition per day) for untreated (PBS), METH, OVA, METH + OVA, LPS, and METH + LPS and error bars indicate STDEV. Symbols (*, π, ϕ, λ, #, σ, and ψ) indicate *P* value significance (*P* < 0.05) calculated using ANOVA and adjusted by use of the Tukey’s post-hoc analysis. *, π, ϕ, and λ indicate significantly higher CD4^+^ cell infiltration than in the untreated, METH, OVA, and METH + OVA-treated groups, respectively. #, σ, and ψ indicate significantly lower CD4^+^ cell infiltration than in the untreated, OVA, and LPS-treated groups, respectively. Representative CD4 stained tissue sections of **(C)** lungs and **(D)** spleens excised from untreated, METH, OVA, METH + OVA, LPS, and METH + LPS-treated animals after 7 days post-Ag challenge are shown (scale bars for 10 and 60 × magnification, 100 μm). Brown staining indicates CD4^+^ cellular infiltration.

We and others have shown that METH reduces T cell populations and their proliferation in mice^13,21,24^. METH also negatively impacts the T cell-mediated immune response affecting microbial control^20,24^. Moreover, METH enhances T lymphocyte apoptosis^15^. Here, we assessed the effect of METH and TD/TI Ag sensitization on helper T cell migration to pulmonary and splenic tissue using immunohistochemistry (IHC; Fig. 3) with CD4^+^ specific Ab. Using microscopy, we counted the number of CD4^+^ T cells in the lungs (Fig. 3A) and spleens (Fig. 3B) of untreated, METH, OVA, METH + OVA, LPS, and METH + LPS-treated mice 3- and 7-days post-Ag administration. On day 3 post-Ag challenge, the lungs of METH-treated animals had higher number of CD4^+^ lymphocytes compared to rodents in all the other experimental groups (*P* < 0.05; Fig. 3A). LPS-sensitized mice also showed lower number of helper T cells compared to animals treated with METH + OVA (*P* < 0.05). Similarly, mice treated with METH + LPS displayed higher pulmonary CD4^+^ T cell infiltration than OVA- and LPS-challenged animals (*P* < 0.05). Seven days post-Ag administration, OVA-treated mice had the highest CD4^+^ T lymphocyte recruitment to the lungs followed by untreated control animals (Fig. 3A). In addition, LPS-sensitized rodents exhibited lower and higher CD4^+^ T cell pulmonary recruitment than METH-treated animals challenged with either Ag (*P* < 0.05).

In splenic tissue, METH-treated animals evinced the highest helper T lymphocyte recruitment (Fig. 3B) 3 days post-Ag administration. METH-treated mice challenged with OVA and LPS had significantly higher numbers of CD4^+^ T cells in splenic tissue than OVA-treated groups (*P* < 0.05). Likewise, spleens excised from METH + LPS mice demonstrated higher splenic lymphocytic infiltration than animals in METH + OVA and LPS groups (*P* < 0.05). On day 7 post-Ag administration, spleens removed from METH-treated animals challenged with OVA or LPS displayed lower CD4^+^ T cell infiltration than untreated or Ag-treated mice (*P* < 0.05; Fig. 3B).

IHC in the lungs of untreated, METH, METH + OVA, and METH + LPS-treated mice revealed weak immunoreactivity to CD4^+^ T lymphocytes 7 days post-antigenic challenge (Fig. 3C). Animals challenged with OVA and LPS displayed CD4^+^ T lymphocytes scattered throughout interstitial pulmonary tissue (Fig. 3C). In splenic tissue, untreated, OVA-, and LPS-treated mice showed abundant CD4^+^ T cell immunoreactivity 7 days post-antigenic administration (Fig. 3D). Untreated rodents demonstrated prominent CD4^+^ T cell presence in the periarteriolar sheath, throughout lymphoid follicles, and in the red pulp (Fig. 3D). Animals sensitized with OVA evinced CD4^+^ T lymphocyte aggregation in the splenic marginal and mantle zone of lymphoid follicles (Fig. 3D). Similarly, the spleens of LPS-treated mice exhibited CD4^+^ T cells at the mantel zone forming a cradling-like pattern around the germinal center (Fig. 3D). All the METH-treated groups demonstrated weak immunoreactivity against CD4^+^ T lymphocytes (Fig. 3D).

**Figure 4:**
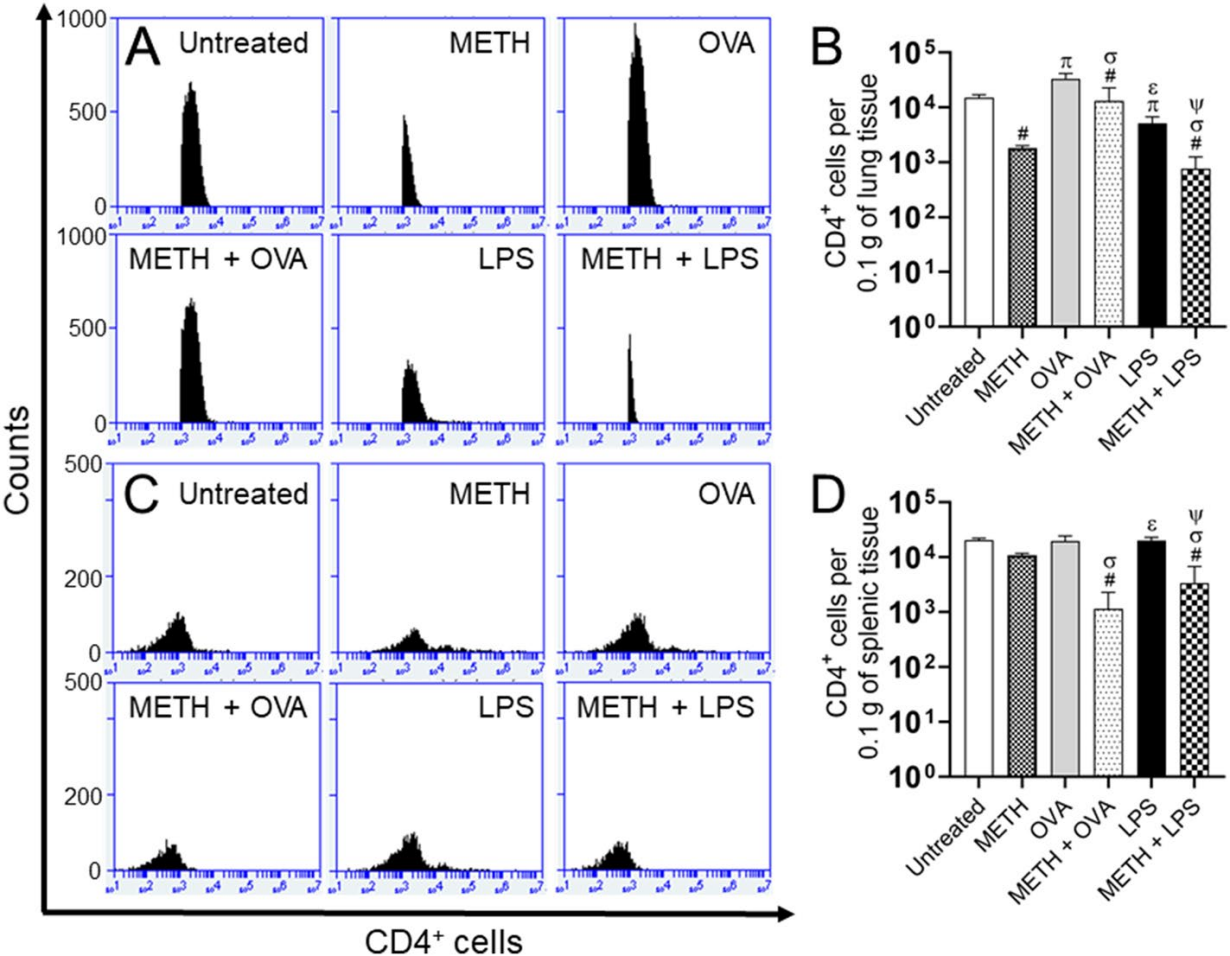
Administration of METH reduces the infiltration of CD4^+^ T cells to the lungs and spleen of rodents challenged with OVA or LPS. **(A,B)** Pulmonary and **(C,D)** splenic tissue was excised (*n* = 5 animals per group), 0.1 g weighed, and homogenized at 7 post-antigenic challenge. Samples were stained with fluorescence-labeled monoclonal antibodies (mAb) for CD4^+^ T cells and analyzed using flow cytometry. Representative plots are shown. Each plot was generated after 10,000 events were analyzed. For each graph, bars represent the mean values; error bars indicate standard deviations. Symbols (π, ε, #, σ, and ψ) indicate *P* value significance (*P* < 0.05) calculated using ANOVA and adjusted by use of the Tukey’s post-hoc analysis. π and ε indicate significantly higher CD4^+^ cell infiltration than in the METH and METH + OVA-treated groups, respectively. #, σ, and ψ indicate significantly lower CD4^+^ cell infiltration than in the untreated, OVA, and LPS-treated groups, respectively. The experiments were performed twice and similar results were obtained.

Corresponding flow cytometry analyses of pulmonary (Fig. 4A,B) and splenic (Fig. 4C,D) tissues excised from rodents 7 days post-Ag challenge validated that METH significantly impairs CD4^+^ T lymphocyte infiltration.

Our results revealed an early increase in helper T cell recruitment to pulmonary and splenic tissue in METH-treated animals followed by a considerable reduction of these lymphocytes indicating a possible increased susceptibility to microbial disease in METH users.

### METH reduces CD8^+^ lymphocyte recruitment into pulmonary and splenic tissue of C57BL/6 mice after Ag challenge

**Figure 5:**
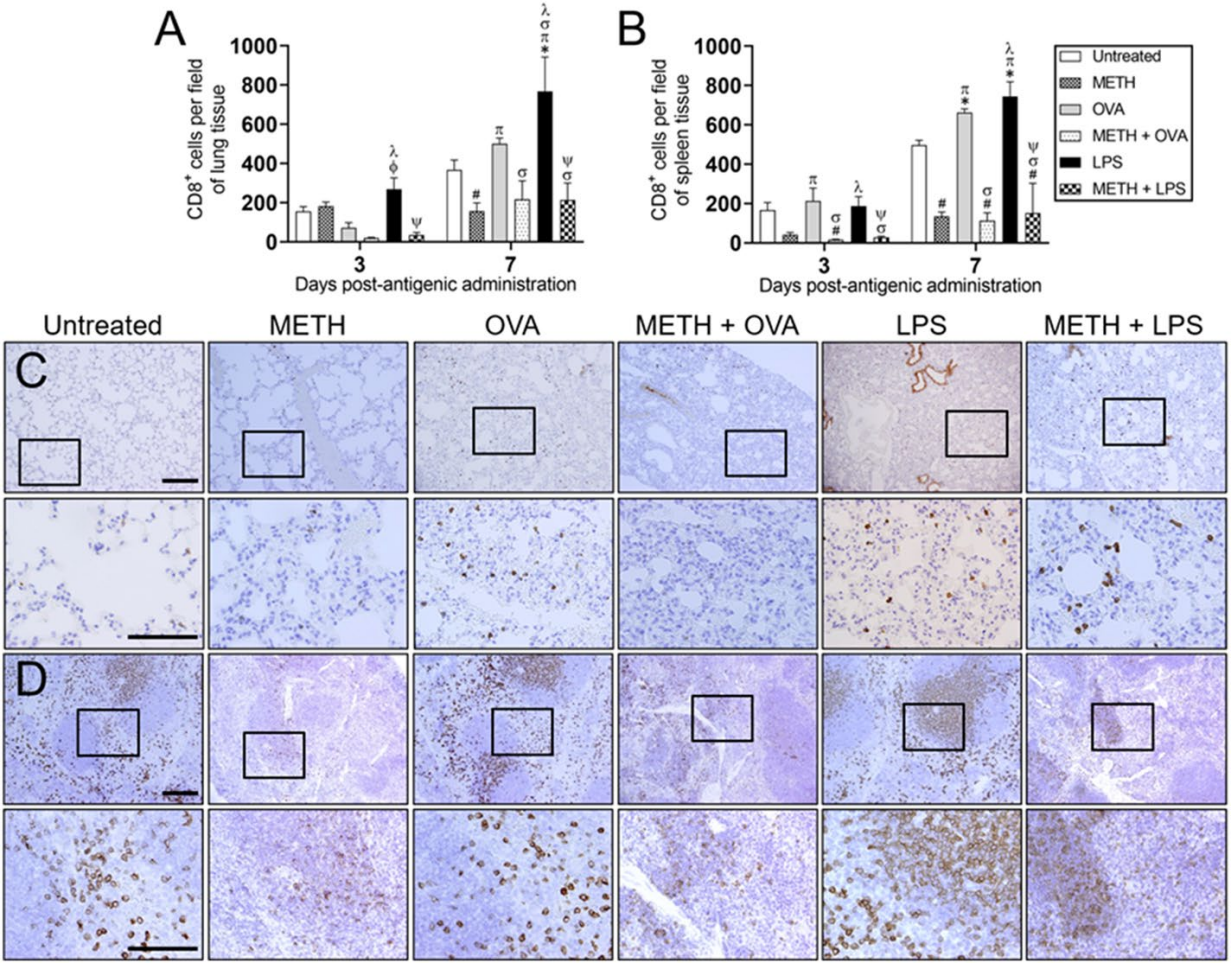
METH decreases CD8^+^ cell recruitment into murine tissues after OVA or LPS challenge. Counts of cytotoxic T (CD8^+^) cells per 0.1 g of **(A)** lung and **(B)** splenic tissue of C57BL/6 mice (*n* = 5 mice per condition per day) 3- and 7-days post-METH and OVA/LPS administration were analyzed by microscopic counts. Bars indicate the average number of CD8^+^ cells (*n* = 5 mice per condition per day) for untreated (PBS), METH, OVA, METH + OVA, LPS, and METH + LPS and error bars indicate STDEV. Symbols (*, π, ϕ, λ, #, σ, and ψ) indicate *P* value significance (*P* < 0.05) calculated using ANOVA and adjusted by use of the Tukey’s post-hoc analysis. *, π, ϕ, and λ indicate significantly higher CD8^+^ cell infiltration than in the untreated, METH, OVA, and METH + OVA-treated groups, respectively. #, σ, and ψ indicate significantly lower CD8^+^ cell infiltration than in the untreated, OVA, and LPS-treated groups, respectively. Representative CD8 stained tissue sections of **(C)** lungs and **(D)** spleens excised from untreated, METH, OVA, METH + OVA, LPS, and METH + LPS-treated animals after 7 days post-Ag challenge are shown (scale bars for 10 and 60 × magnification, 100 μm). Brown staining indicates CD8^+^ cellular infiltration.

CD8^+^ or cytotoxic T cells are essential for immune defense against intracellular microorganisms, including viruses and bacteria, and for tumor surveillance. CD8^+^ T cells recognize Ag from infected or malignant cells presented on MHC class I molecules, become activated. They can secrete cytokines, primarily TNF-α and IFN-γ, which have anti-tumor and anti-viral effects. These lymphocytes can also produce and release cytotoxic granules such as perforins and granzymes to kill virally infected or tumorigenic cells. In addition, cytotoxic lymphocytes can directly destroy infected cells via Fas/FasL interactions resulting in the activation of the caspase cascade resulting in apoptosis. Given that METH users are susceptible to viral infections due to their risky behavior and cytotoxic T lymphocytes are important in the control of these infections, we explored the impact of this psychostimulant on CD8^+^ T cell recruitment to pulmonary and splenic tissues. Microscopic counts of CD8^+^ lymphocytes in the pulmonary (Fig. 5A) and splenic (Fig. 5B) tissue of each experimental group was performed 3- and 7-days post-Ag administration. LPS-treated mice evinced significantly higher recruitment of CD8^+^ lymphocytes to their lungs than the OVA, METH + OVA, and METH + LPS groups 3 days-post Ag challenge (*P* < 0.05; Fig. 5A). Moreover, LPS-treated mice had the highest CD8^+^ T cell infiltration to the lungs on day 7 post-Ag sensitization (*P* < 0.05). Similarly, mice challenged with OVA had higher pulmonary cytotoxic cell infiltration than each METH-treated group (*P* < 0.05). Excised lung tissue from METH-treated rodents exhibited lower cytotoxic lymphocyte recruitment than untreated controls (*P* < 0.05; Fig. 5A).

In splenic tissue, untreated, OVA, and LPS-treated mice showed significantly higher CD8^+^ cell infiltration than METH + OVA animals 3 days post-Ag administration (*P* < 0.05). Likewise, spleens from mice sensitized with OVA had significantly higher cytotoxic lymphocyte recruitment than METH and METH + LPS-treated groups (*P* < 0.05). In addition, METH + LPS-treated animals displayed lower CD8^+^ T cell infiltration to splenic tissue than LPS-treated animals 3 days-post Ag challenge (*P* < 0.05; Fig. 5B). Furthermore, we observed considerable reduction of cytotoxic cellular infiltration in all the mice groups treated with METH 7 days post-Ag challenge (*P* < 0.05; Fig. 5B). LPS-treated spleens experienced the highest CD8^+^ T cell recruitment (*P* < 0.05). In fact, cytotoxic cell recruitment in splenic tissue of animals followed this order: LPS > OVA > untreated mice (*P* < 0.05; Fig. 5B).

On day 7 post-Ag administration, IHC of pulmonary tissues excised from untreated, OVA, and LPS-treated C57BL/6 mice resulted in positive brown immunostaining for a CD8-specific antibody scattered throughout inflammatory infiltrates in interstitial tissue (Fig. 5C). In contrast, the lungs of animals in the METH-, METH + OVA-, and METH + LPS-groups showed negative immunostaining against CD8^+^ T cells (Fig. 5C). Similarly, IHC of the spleens excised from untreated, OVA, and LPS-treated mice demonstrated moderate to strong membranous immunoreactivity against CD8^+^ lymphocytes in the rim of lymphoid follicles and interfollicular space (Fig. 5D). However, each of the groups treated with METH revealed weak immunostaining against CD8^+^ T cells, limited to the rim of lymphoid follicle and to the interfollicular space (Fig. 5D).

**Figure 6:**
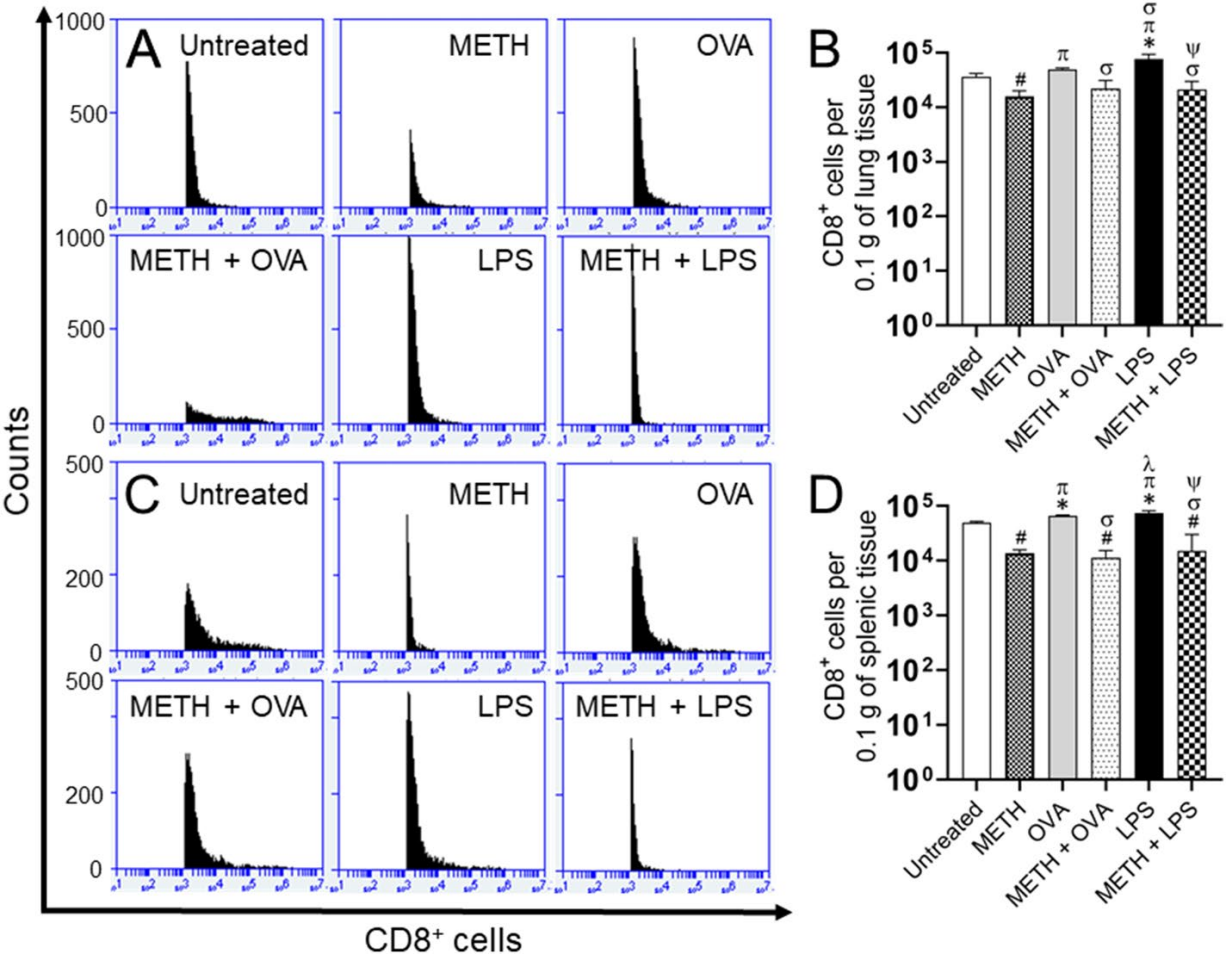
METH injection reduces the infiltration of CD8^+^ T cells to the lungs and spleen of mice sensitized with OVA or LPS. **(A,B)** Pulmonary and **(C,D)** splenic tissue was excised (*n* = 5 animals per group), 0.1 g weighed, and homogenized at 7 post-antigenic challenge. Samples were stained with fluorescence-labeled mAb for CD8^+^ T cells and analyzed using flow cytometry. Representative plots are shown. Each plot was generated after 10,000 events were analyzed. For each graph, bars represent the mean values; error bars indicate standard deviations. Symbols (*, π, ϕ, λ, #, σ, and ψ) indicate *P* value significance (*P* < 0.05) calculated using ANOVA and adjusted by use of the Tukey’s post-hoc analysis. *, π, and λ indicate significantly higher CD8^+^ cell infiltration than in the untreated, METH, and METH + OVA-treated groups, respectively. #, σ, and ψ indicate significantly lower CD8^+^ cell infiltration than in the untreated, OVA, and LPS-treated groups, respectively. The experiments were performed twice and similar results were obtained.

Quantitative analysis on tissues removed from C57BL/6 mice 7 days post-Ag sensitization using flow cytometry (Fig. 6) confirmed that METH significantly reduces CD8^+^ T cell recruitment into their lungs (Fig. 6A,B) and spleen (Fig. 6C,D).

These results demonstrate that METH impairs CD8^+^ T cell responses in pulmonary and splenic tissue and suggest that METH consumption may increase users’ susceptibility to viral infections.

### METH compromises the expression of CD3 and CD28 molecules on T cells

**Figure 7:**
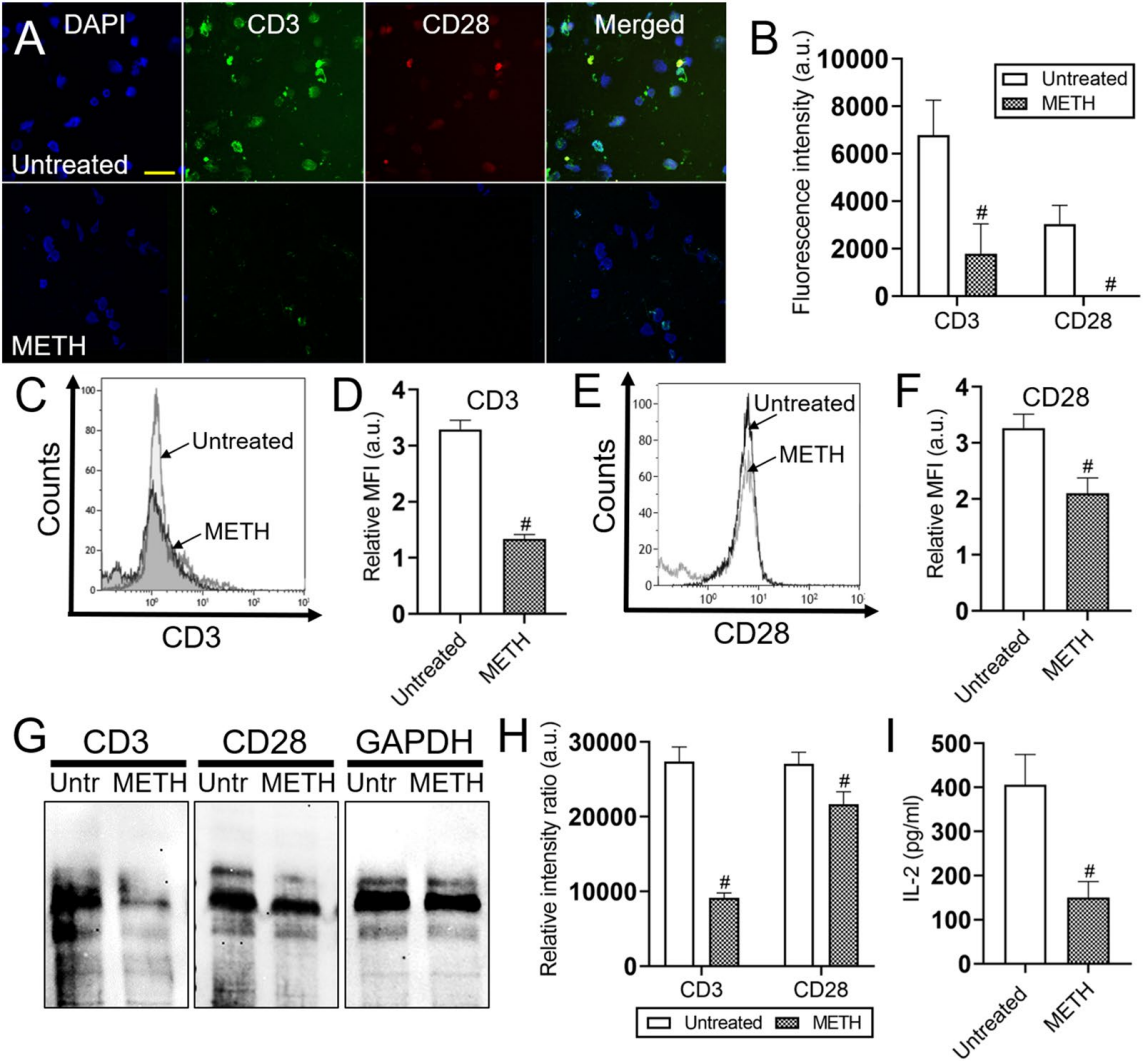
METH reduces the distribution and expression of CD3 and CD28 molecules on the surface of human Jurkat T cells. **(A)** Immunofluorescent images show the distribution of CD3 (green; anti-CD3 conjugated to Alexa Fluor 488) and CD28 (red; anti-CD28 conjugated to APC) on the surface of untreated (upper panel) and METH-treated (lower panel) Jurkat T cells. Nuclei of T cells were stained in blue with dapi. Scale bar, 20 μm. **(B)** The CD3 and CD28 fluorescent intensity of untreated and METH-treated Jurkat T cells was analyzed. Bars represent the mean of 10 cell measurements and error bars indicate STDEV. The number sign (#) denotes *P* value significance (*P* < 0.05) calculated using student’s *t*-test analysis. The mean fluorescence intensity (MFI) of **(C,D)** CD3 and **(E,F)** CD28 molecules on Jurkat T cells was analyzed by flow cytometry. A density of 2 × 10^5^ Jurkat T cells was treated with 25 μM METH for 2 h. Representative histograms of untreated and METH-treated Jurkat T cells are shown. Each plot was generated after 10,000 events were analyzed. The relative MFI of IgM receptor was analyzed. **(G)** The expression of CD3 and CD28 in Jurkat T cells was determined by western blot analysis. T cells were cultured with 25 μM METH for 2 h. GAPDH was used as a housekeeping gene control. Both conditions (Untreated and METH) were run in the same gel next to each other and processed in parallel. We cropped each gel to improve the clarity and conciseness of the presentation. Original full-length gels are presented in Supplementary (S) Fig. 1A (CD3), SFig. 1B (CD28), and SFig. 1C (GAPDH). **(H)** The levels of expression of CD3 and CD28 were measured by determining the relative intensity ratios. Individual band intensities from the western blot were quantified using ImageJ software. The GAPDH gene was used as a reference to determine the relative intensity ratios shown in panel **H**. **(I)** Twenty-four hours culture supernatants from untreated and METH-treated Jurkat T cells stimulated with a mixture of 50 ng/ml phorbol myristate acetate (PMA), 1 μg/ml ionomycin, and 30 μg/ml phytohemagglutin-L (PHA) were analyzed for IL-2 production by ELISA. For graphs in **D**,**F**,**H**, and **I**, bars represent the mean of 3 independent experiments (*n* = 3) and error bars indicate STDEV. The number sign (#) indicates *P* value significance (*P* < 0.05) calculated using student’s *t*-test analysis.

Human Jurkat T cells express CD3 and CD28 on their surface. The two-signal theory suggests that T cell activation requires both Ag recognition via the TCR-CD3 complex and additional costimulatory signals derived from CD28 and other receptors^19^. Given that METH modified CD4 and CD8 T cell responses to Ag stimulation in vivo, we investigated the effects of the drug on the distribution and expression of CD3 and CD28 on the surface of Jurkat T cells using fluorescent microscopy (anti-CD3-conjugated to Alexa Fluor 488, green; anti-CD28-conjugated to APC, red; dapi, blue) (Fig. 7A). The quantification of fluorescent CD3 and CD28 revealed that Jurkat T cells treated with METH had a substantial reduction of these receptors distributed on their surface relative to untreated cells (*P* < 0.05; Fig. 7B). Flow cytometry analyses validated that METH significantly decrease the distribution of CD3 (*P* < 0.05; Fig. 7C,D) and CD28 (*P* < 0.05; Fig. 7E,F) molecules on Jurkat T cells compared to control cells. We also assessed the impact of METH on Jurkat T CD3 and CD28 expression and demonstrated that METH considerably impaired the expression of these molecules (*P* < 0.05; Fig. 7G,H). Since IL-2 is an important modulator of cell-mediated immunity, we examined the impact of METH on Jurkat T cell production of this cytokine after Ag stimulation. Supernatants of METH-treated Jurkat T cell cultures had significantly lower IL-2 levels than untreated lymphocytes (*P* < 0.05; Fig. 7I). Hence, our findings suggest that METH interferes with the development of the cellular immunity, making users vulnerable to infectious diseases.

## Discussion

We explored the impact of prolonged administration and increasing doses of METH on murine pulmonary tissue architecture after exposure to TD and TI Ag, OVA and LPS, respectively. We found that METH administration resulted in partial collapse of regions of the lungs in mice. METH has been previously described as an illicit drug that causes thoracic disease^25^. Pulmonary complications of METH use include non-cardiac pulmonary oedema, acute respiratory distress syndrome, alveolar hemorrhage, pneumonia, and pneumoconiosis^26^. For example, a patient with 15- to 20-year history of almost daily recreational inhalation of METH presented interstitial pulmonary fibrosis^27^. Individuals deceased from acute METH intoxication have demonstrated enlarged cardiothoracic ratio^28^. Acute inhalation exposure to vaporized METH results in elevated free radical production and lung injury in mice^29^. Similarly, METH-treated mice challenged with Ag displayed considerable leukocyte infiltration, congestion of the interstitial capillaries, and coagulative necrosis. METH induces massive infiltration of macrophages and neutrophils in response to Ag sensitization and prolonged presence of these leukocytes may result in considerable collateral tissue damage via increased oxidative stress. We observed that METH causes apoptosis in pneumocytes of the respiratory system. METH-induced apoptosis of alveolar epithelium cells is mediated by endoplasmic reticulum stress^30^, resulting in the reduction of pulmonary alveoli. Concurrence of autophagy with apoptosis in alveolar epithelial cells contributes to chronic pulmonary toxicity induced by chronic METH use^31^. Also, we previously demonstrated that pharmacological levels of METH in human blood and organs are cytotoxic to ∼ 20% of the phagocytes^32^. Another pulmonary complication of METH use is an increase in the permissiveness to opportunistic microorganisms. We have previously shown that METH administration enhances histoplasmosis, a systemic mycosis caused by *Histoplasma capsulatum*, in mice by increasing pulmonary fungal burden and host inflammation^24^. METH promotes the AIDS-related fungus *Cryptococcus neoformans* dissemination from the respiratory tract into the brain parenchyma of rodents, facilitating fungal meningitis^20^. Also, a study of HIV-infected patients in Thailand reported that 40% of those also infected with *Mycobacterium tuberculosis* had a history of METH use^10^.

In contrast, mice treated with METH and challenged with OVA demonstrated reduced inflammation and lung injury. It is conceivable that OVA stimulate T lymphocyte recruitment, inhibiting neutrophil infiltration and preventing tissue damage in METH users. Since the regulation of Th17/Treg function contributes to the attenuation of chronic airway inflammation in OVA-induced murine asthma model by several compounds^33–35^, it is possible that METH promotes the activation of these subset of T cells and modulates neutrophilic infiltration to pulmonary tissue. For example, PAP-1, a selective inhibitor of Kv1.3 channel in lung tissues, inhibit the ERK-NF-κB signaling pathway, which is a mechanism to reduce airway inflammation in case of OVA-LPS-induced neutrophilic asthma^36^. Future studies are needed to elucidate the role of TD Ag and T cell regulation on METH-mediated inflammation, especially in chronic users.

We also examined the effects of chronic-like METH administration on the anatomy of the spleen after sensitization with TD and TI Ag. METH-treated mice demonstrated substantial splenic tissue injury that was exacerbated by Ag exposure. Excised spleens from prolonged METH-treated animals showed significant splenocyte apoptosis. METH accumulates extensively and has intermediate clearance in the spleen of humans^12^ and rodents^37^. We have previously shown that METH stimulates the infiltration of elevated numbers of macrophages and neutrophils in the spleen^21^, which may also be associated with elevated free radical formation^29^ that can promote the significant necrosis we identified. Additionally, increased quantities of pro-inflammatory cytokines, IFN-γ, TNF-α, IL-6, and IL-12, previously reported correlate with a sustained cellular inflammatory response and tissue injury^21^. Although METH-induced apoptosis in the spleen of rats has been previously shown by Iwasa and colleagues^14^, they used a single injection of 25 mg/Kg METH, which is an extremely high dose or 2.5 times higher than the dose we used in our study. Unpublished studies in our laboratory showed that a single peritoneal administration with 20 mg/Kg of METH to C57BL/6 mice is lethal in some of the injected animals. Furthermore, Sprague–Dawley rats seems to be more susceptible than mice to multiple injections with similar doses.

We investigated the impact of METH on cell-mediated immune responses in murine pulmonary and splenic tissue after exposure to OVA and LPS. Pulmonary and splenic tissue exposed to METH had a significant surge of CD4^+^ T cell infiltration after 3 days post-drug injection but their recruitment dramatically plummeted 7 days-post METH administration even in mice challenged with Ag. We observed a substantial reduction in cytotoxic T cell infiltration into the lungs and spleens of METH-treated mice with or without Ag challenge. Previous studies have demonstrated that METH reduces CD8^+^ T lymphocytes in the thymus^17^ and spleen^16^. A previous study that adopted our mouse model of prolonged METH administration^24^ showed a reduction in both helper and cytotoxic T lymphocytes in the spleen of drug exposed animals using flow cytometry suggesting a decrease in Ag presentation and an increase in cell death in these populations^16^. Since METH administration also decreases the number and responsiveness of innate natural killer cells, a reduction in adaptive cytotoxic T cells might leave the host without defense against commonly METH-associated viral infections.

Studies using human Jurkat T cells and performing three complementary, but different experimental methods showed that METH impairs the expression and distribution of CD3 and CD28 on their surface. Utilizing flow cytometry and IHC analyses, we previously demonstrated that METH substantially reduces T cells (CD3^+^) in pulmonary and splenic tissues, providing a strong evidence supporting these in vitro results^21^. METH also significantly reduces the production of IL-2^17^, a cytokine involved in the amplification of cell-mediated immunity. It is plausible that METH compromises the activation of T cells and this might explain the considerable decreased in tissue recruitment. We have previously shown that METH induces T cells to produce large amounts of IL‐4 and IL‐10^24^, and the secretion of these cytokines contributes to the inhibition of T cell proliferation^38^ after Ag exposure. METH interferes with T cell lymphocyte proliferation upon Ag challenge^24^. METH exposure modifies T cell cycle entry and progression^39^, alters mitochondrial function resulting in oxidative damage^15^, and causes apoptosis^14^. Our study provides additional evidence supporting that METH use might be associated with higher viral loads and lower CD4^+^ and CD8^+^ T cell counts in HIV-infected individuals^40^.

Our results demonstrate that METH causes formidable morphological changes to the lungs and spleen of mice including apoptosis, which may have important implications in combating common infections in illicit drug users. METH reduces T cell populations in pulmonary and splenic tissue, providing additional evidence to the susceptibility of users to HIV infection and disease as well as to the acquisition of AIDS-associated secondary infections. METH inhibits T lymphocyte activation and proliferation by interfering with costimulatory molecules and IL-2 production. Future investigations should focus on elucidating how METH interferes with T cell costimulatory pathways and understanding how METH inhibits T cell receptors on T cells bind to Ag-complexed class II MHC molecule on the APC surface during T cell activation. Despite the importance of cytotoxic T cells in the fight against viruses, only a few studies are available addressing the impact of METH on CD8^+^ T cell effector functions and the outcomes of cytotoxic T lymphocyte interactions with METH-associated viral infections. We anticipate that our findings will result in a deeper understanding of the mechanisms for the increased susceptibility to microbial disease in METH abusers.

## Materials and methods

### Mouse model of increasing and prolonged doses of METH administration

High‐dose METH users initially take small amounts of the drug intermittently before progressively increasing the dose^41^. To simulate this pattern, increasing doses (2.5, 5, and 10 mg/kg/day on weeks 1, 2, and 3, respectively) of METH (Sigma) were intraperitoneally (i.p.) administered daily to female C57BL/6 mice (age, 6–8 weeks; Charles Rivers) over 21 days. Phosphate‐buffered saline (PBS; untreated)-treated animals were used as controls. Mice that received the daily injection of METH lost approximately 2 g of body weight, compared with control mice^24^. After PBS or METH treatments, animals were anesthetized with 100 mg/kg ketamine (Keta-set; Henry Schein) and 10 mg/kg xylazine (Anased; Henry Schein) cocktail and intranasally (i.n.) challenged with a single dose of 50 μg/ml of either OVA (main protein found in egg white; Sigma) or LPS (outer membrane component of Gram negative bacteria; Sigma) in a 50 μl suspension. Hence, we had six experimental groups of mice that were randomly distributed: untreated, METH, OVA, METH + OVA, LPS, and METH + LPS. On days 3 and 7 post-Ag challenge, each mouse was anesthetized and euthanized. Then, the organs were removed to analyze the role of METH on T cell responses. The right and left lungs of each mouse were weighed and collected for flow cytometry and histology, respectively. Each spleen was divided into two similar size halves and each half of tissue was weighed and utilized for flow cytometry and histology. Naïve mice were also used as controls. All the untreated and METH-treated animals survived the injection regime and Ag challenge. Mice were maintained in an environment with an ambient temperature between 22–24.5 °C with a 12:12-h light/dark cycle and ad libitum access to food and water.

### Ethics statement

All animal studies were conducted according to the experimental practices and standards approved by the Institutional Animal Care and Use Committee at the NYIT College of Osteopathic Medicine (Protocol #: 2016-LRM-01). This study was carried out in compliance with the ARRIVE guidelines^42^.

### Rationale for METH doses used in mice

The concentrations of METH used in the experiments are physiologically relevant. Controlled studies have indicated that a single 260 mg dose peaks at a level of 7.5 μM^43^. A single dose of 260 mg would be expected to produce 7.5 to 28.8 μM blood METH levels. Binge doses of 260 to 1,000 mg produce 17 to 80 µM blood METH levels and levels in the µM range of hundreds in organs, including the spleen^37^. Therefore, we selected 2.5 to 10 mg of METH/kg/day to perform our in vivo experiments^16,24^ and 25 µM of METH to perform our in vitro experiments^24^.

### Histology

After METH administration and Ag sensitization, each mouse was euthanized and lung/spleen tissues were excised and fixed in 4% paraformaldehyde (Sigma) for 24 h. Tissues were processed, embedded in paraffin, and 4 μm sagittal sections were fixed to glass slides. The tissues were then subjected to hematoxylin and eosin (H&E) staining, CD4- (clone W3/25)^44^, and CD8- (clone 32-M4)^45^ specific antibody (Ab; conjugated to horseradish peroxidase; dilution: 1:1000; Santa Cruz Biotechnology; SCB) immunostaining to assess tissue morphology, helper T cell, and cytotoxic T cell infiltration (brown), respectively. The slides were visualized using an Axiovert 40 CFL inverted microscope (Carl Zeiss; CZ), and images were captured with an AxioCam MRc digital camera using the Zen 2011 digital imaging software. The hyperplasia of type II pneumocytes was recorded microscopically in single cuboidal cells showing large nuclei, prominent nucleoli, and scant to abundant cytoplasm. The infiltration of CD4^+^ and CD8^+^ cells to pulmonary and splenic tissue was quantified by cell counts using the recorded 40 × images. Each image was blindly analyzed by three independent investigators. Ten microscopic fields per image were counted and the average was calculated per image (*n* = 10 images; 10 fields per image; 2 images per mouse; 5 mice per condition).

### Lung injury score

The lung injury score (LIS) was quantified by two investigators blinded to the treatment groups using published criteria by the American Thoracic Society^46^. The LIS was obtained by the sum of each of the following six independent variables: neutrophils in the alveolar spaces and/or in the interstitial spaces, presence of hyaline membranes, proteinaceous debris filling the airspaces, alveolar septal thickening, and alveolar congestion. This sum was weighted according to the relevance ascribed to each feature by the American European Consensus Committee^46^ and then was normalized to the number of fields evaluated and arbitrarily multiplied by 10 to obtain continuous values between zero and one (both inclusive). The resulting LIS was derived from the following calculation: LIS = {[(20 × A) + (14 × B) + (6 × C) + (6 × D) + (2 × E) + (2 × F)]/(number of fields × 100) × 10}.

### TUNEL assay

Paraffin-embedded slides from lung tissue were prepared as described and analyzed for DNA strand breaks by fluorescent enzymatic labeling of the free 3′OH termini of modified nucleotides using a commercially available kit (In Situ Cell Death Detection Kit, Fluorescein; Roche). The slides were visualized with a Zeiss LSM 700 confocal laser scanning microscope (CZ) set at excitation/emission wavelengths of 488/510 nm and at a magnification of 40 × . Images were collected using an AxioCam digital camera and analyzed using Zen Lite digital imaging software (CZ). For each staining, images were blindly analyzed by three independent investigators. Ten microscopic fields per image were counted and the average was calculated per image (*n* = 10 images; 10 fields per image; 2 images per mouse; 5 mice per condition).

### Spleen injury score

The degree of splenic injury was evaluated with a semiquantitative scoring system by two investigators blinded to the treatment groups^47^. The evaluation criteria used was the following: 0, the morphology of spleen white pulp was obvious, which was characterized by obvious per arterial lymphoid sheath, gerontology center, capsule zone and marginal zone; 1, mild disorder of spleen white pulp, characterized by local hyperplasia; 2, moderate disorder of spleen white pulp, blurring of the boundary between white pulp and red pulp; 3, high disorder of spleen white pulp, almost no significant difference between white pulp and red pulp.

### Splenocyte viability assay

We assessed whether METH induces apoptosis in splenocytes, impairing their viability. Apoptotic splenocytes were analyzed by flow cytometry using an annexin V-FITC and PI kit (Becton Dickinson; BD). Splenocytes isolated from 0.1 g of excised and homogenized tissue in 1 ml PBS from PBS- and METH-treated C57BL/6 mice were transferred to a flow cytometry tube, washed twice with cold PBS, and suspended in binding buffer. Each sample was processed similarly as previously described^32^. Five microliters each of annexin V-FITC and PI was added to cells. Each suspension was gently vortexed and incubated for 15 min at room temperature (25 °C) in the dark. Then 400 μl of binding buffer was added to each tube and samples were analyzed (10,000 events per sample) by flow cytometry within 1 h using a BD Accuri C6 flow cytometer. The percentage of cells positive for PI (red) and/or annexin V-FITC (green) was determined using the FCS Express software.

### Measurement of CD4 and CD8 T cell recruitment in murine tissue using flow cytometry

For flow cytometry staining, primary cells were isolated from 0.1 g of excised and homogenized lung and spleen tissue in 1 mL PBS from 5 mice treated with PBS, METH, OVA, METH + OVA, LPS, and METH + LPS as described above; the cells were washed and then stained with either CD4- (dilution: 1:1000; SCB)-allophycocyanin (APC)-labeled Ab^44^, CD8- (dilution: 1:1000; SCB)- peridinin chlorophyll protein-cyanide (PerCP-Cy5.5)-labeled Ab^45^ or their corresponding isotype controls. Each sample was processed on a BD Accuri C6 flow cytometer, analyzed using the FCS Express software version 4, and the number of CD4^+^ and CD8^+^ cells per 0.1 g of tissue was reported per condition.

### Human Jurkat T lymphocyte leukemia cell lines

Jurkat clone E6-1 (American Type Culture Collection; ATCC) cells were cultured in RPMI 1640 medium (HyClone) and supplemented with 15% heat-inactivated fetal calf serum (FCS; Atlanta Biologicals), 100 U/ml penicillin (Gibco), 100 µg/ml streptomycin (Gibco), and 0.25 µg/ml amphotericin B (Gibco). Exponentially growing cells, at approximately 60–75% confluence, were counted, and seeded into 24-well microtiter plates (Corning). The incubation conditions of the cells were 37 °C in a humidified 5% CO_2_ atmosphere. For each experiment, 2 × 10^5^ Jurkat T cells were cultured with 25 µM METH or without for 2 h and washed 3X with PBS. Then, fluorescent microscopy, flow cytometry, and western blot analyses were performed.

### Fluorescent microscopy

Monolayers of Jurkat T cells were fixed on a glass-bottom petri dish (TF) with 4% formaldehyde (TF) for 20 min at RT. The cells were washed 3X with 5% Tween 20 (TF) in PBS and blocked with 1% BSA in PBS for 1 h at RT. After blocking, the cells were again washed 3X with 5% Tween 20 in PBS and incubated with anti-CD3 (clone PC3/188A)^48^ and anti-CD28 (clone CD28.2)^49^ conjugated to Alexa Fluor 488 (green; 1:200 dilution; SCB) and Allophycocyanin (APC; red; 1:200 dilution; SCB), respectively, in blocking solution in an orbital shaker (Eppendorf Galaxy 170S) at 150 rpm and 37 °C for 1 h. The samples were washed 3X with blocking buffer and incubated with 4′, 6-diamidino-2-phenylindole (dapi; blue; Thermo Fisher; TF) to stain nuclei for 1 h at 37 °C. The slides were washed 3X with PBS, coverslips were affixed, and each sample was viewed to determine the CD3/CD28 distribution on the surface of Jurkat T cells with a Zeiss LSM 700 Confocal Laser Scanning Microscope (CZ) at a magnification of 40 × . Images were collected using an AxioCam digital camera and analyzed using Zen Lite digital imaging software (CZ). This fluorescent microscopy methodology is a modification of a similar and previously described procedure^50^.

### Determining CD3 and CD28 expression on Jurkat T cells using flow cytometry

Monolayers of 10^5^ Jurkat T cells were grown in a 24-well microtiter plate in the absence or presence of METH (25 µM) for 2 h at 37 °C and 5% CO_2_^50^. For flow cytometry analysis, Jurkat T cells were detached with 0.05% trypsin (Corning) treatment for 1 min followed by centrifugation at 980 rpm for 5 min at RT. Cells were washed 3X with PBS and centrifuged as described above. CD3 Alexa Fluor 488-conjugated monoclonal Ab (1:200 dilution)^48^ and CD28 APC-conjugated monoclonal Ab (1:200 dilution)^49^ were added on 1% BSA in PBS to the cells followed by an incubation at 37 °C for 1 h. Samples were processed (10,000 events per sample) on a Gallios flow cytometer (Beckman Coulter) and CD3 and CD28 expression on Jurkat T cells were analyzed using the Kaluza software (Beckman Coulter).

### Western blot analysis

To further understand the impact of METH on the CD3 and CD28 expression in Jurkat T cells, a density of 2 × 10^5^ Jurkat T cells was incubated in a 24-well microtiter plate with 25 µM METH for 2 h. Then, the cells were washed 3X with PBS. Western blot analysis was conducted using cytoplasmic extracts made with a NE-PER nuclear and cytoplasmic extraction kit (TF), as previously described^50^. The mixture was centrifuged at 10,000 × g for 10 min at 4 °C, and the resulting protein content of the supernatant was determined using the Bradford method, employing a Pierce BCA protein assay kit (TF). Lysates were preserved in a protease inhibitor cocktail (TF) and stored at − 20 °C until use. Extracts were diluted with 2 × Laemmli sample buffer (Bio-Rad) and β-mercaptoethanol (Sigma). The mixture was heated to 90 °C for 5 min. Twenty-three µg of protein were applied to each lane of a gradient gel (7.5%; Bio-Rad). Proteins were separated by electrophoresis at a constant 130 V/gel for 90 min and transferred to a nitrocellulose membrane on the Trans-Blot Turbo Transfer System (Bio-Rad) at 25 V for 7 min. The membranes were blocked with 5% BSA in tris-buffered saline (TBST; 0.1% Tween 20) for 2 h at RT. A primary monoclonal CD3^48^ or CD28^49^-specific Ab (1:100 dilution; SCB) was incubated overnight at 4 °C with TBST (5% BSA). After washing the membranes 3X with TBST for 10 min, a goat anti-mouse IgG (H + L) conjugated to horseradish peroxidase was used as a secondary Ab (1:1000; Southern Biotech) and incubated with TBST (5% BSA) for 1 h at RT. The membranes were washed as described above. Protein bands were measured using the iBright FL100 imaging system (Invitrogen) after staining each membrane with chemiluminescence detection reagents (TF). Quantitative measurements of individual band intensities in western blot analyses for CD3 or CD28 were performed using ImageJ software (NIH). Glyceraldehyde-3-phosphate dehydrogenase (GAPDH; dilution, 1:1,000; BD), a cytoplasmic housekeeping protein, was used as loading controls to determine the relative intensity ratio. This western blot protocol was previously described in^50^ and modified accordingly for this study.

### IL-2 determinations

Jurkat T cells grown with and without 25 µM METH were stimulated to secrete IL-2 with a combination of phorbol myristate acetate (PMA; 50 ng/ml), ionomycin (1 μg/ml), and phytohemagglutin-L (PHA; 1 μg/ml) for 24 h. Culture supernatant aliquots were collected, cell debris removed by centrifugation at 6000 g for 10 min, a tablet of protease inhibitor (Roche) added, and stored at − 80 °C until tested. Samples were tested using the Human IL-2 ELISA kit II (BD). The limit of detection for IL-2 was 1 pg/ml.

### Image presentations and drawings

Each shape (e.g., circles, rectangles, triangles, and arrows) or label was used to provide a clear and concise description of each image and were drawn using Microsoft Office 365 PowerPoint (Seattle, WA).

### Statistical analysis

All data were subjected to statistical analysis using Prism 9.0 (Graph Pad). *P* values for multiple comparisons were calculated by analysis of variance (ANOVA) and adjusted using the Tukey’s multiple comparison test. *P* values for individual comparisons were calculated using student's *t*-test analysis. *P* values of < 0.05 were considered significant.

## Supplementary Information

Supplementary Information 1.
