# Supplementary figures and images for: Methamphetamine facilitates pulmonary and splenic tissue injury and reduces T cell infiltration in C57BL/6 mice after antigenic challenge
Source: Sci Rep. 2021 Apr 15;11:8207. doi: 10.1038/s41598-021-87728-4 (PMC8050260; doi:10.1038/s41598-021-87728-4)

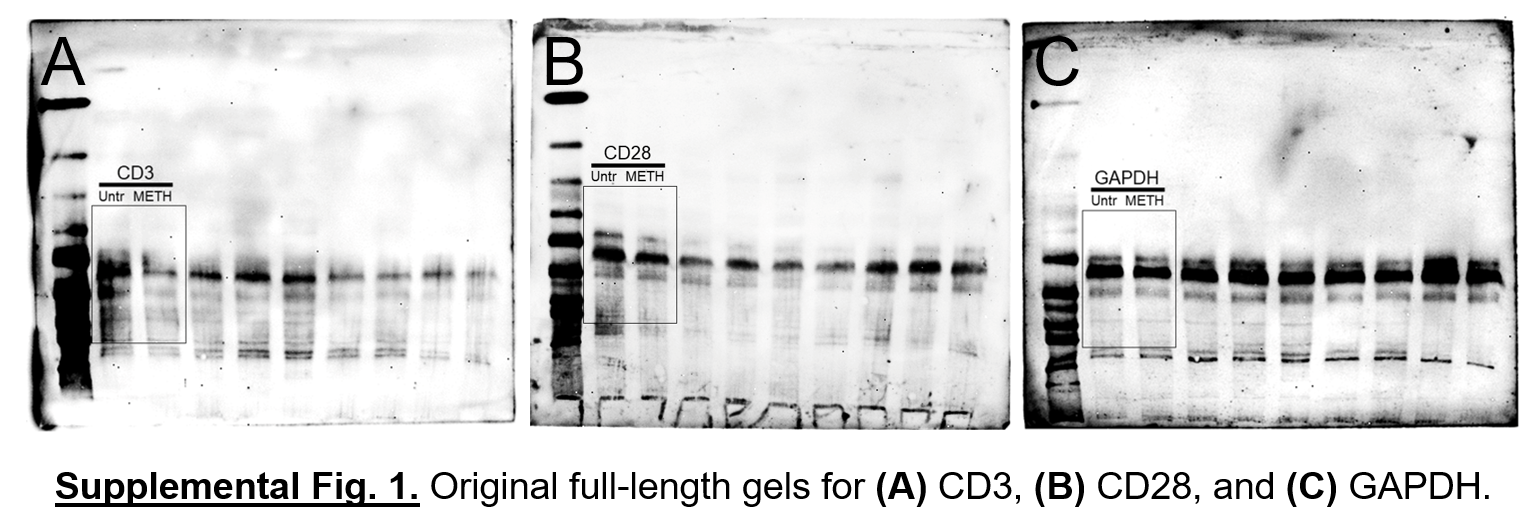

Supplement: Supplementary file 1 — Supplementary Information 1. [file 41598_2021_87728_MOESM1_ESM.tif]
